# Supplementary material for: In Vitro Analysis of LPS-Induced miRNA Differences in Bovine Endometrial Cells and Study of Related Pathways
Source: Animals (Basel). 2024 Nov 22;14(23):3367. doi: 10.3390/ani14233367 (PMC11640166; doi:10.3390/ani14233367)
Supplement: Supplementary file 1 [file animals-14-03367-s001.zip › animals-3229607-supplementary.pdf]

Table S1. Intergroup and intragroup variance analysis of the effects of LPS concentration and time on cell viability.

Tests of Between-Subjects Effects

Dependent Variable: value

Source	Type III Sum of Squares	df	Mean Square	F	Sig.
Corrected Model	1.168[a]	17	.069	35.297	<0.001
intercept	38.373	1	38.373	19706.749	<0.001
time	.385	2	.193	98.900	<0.001
control	.562	5	.112	57.720	<0.001
time * control	.221	10	.022	11.364	<0.001
Error	.070	36	.002		
Total	39.611	54			
Corrected Total	1.239	53			

a. R Squared = .943 (Adjusted R Squared = .917)

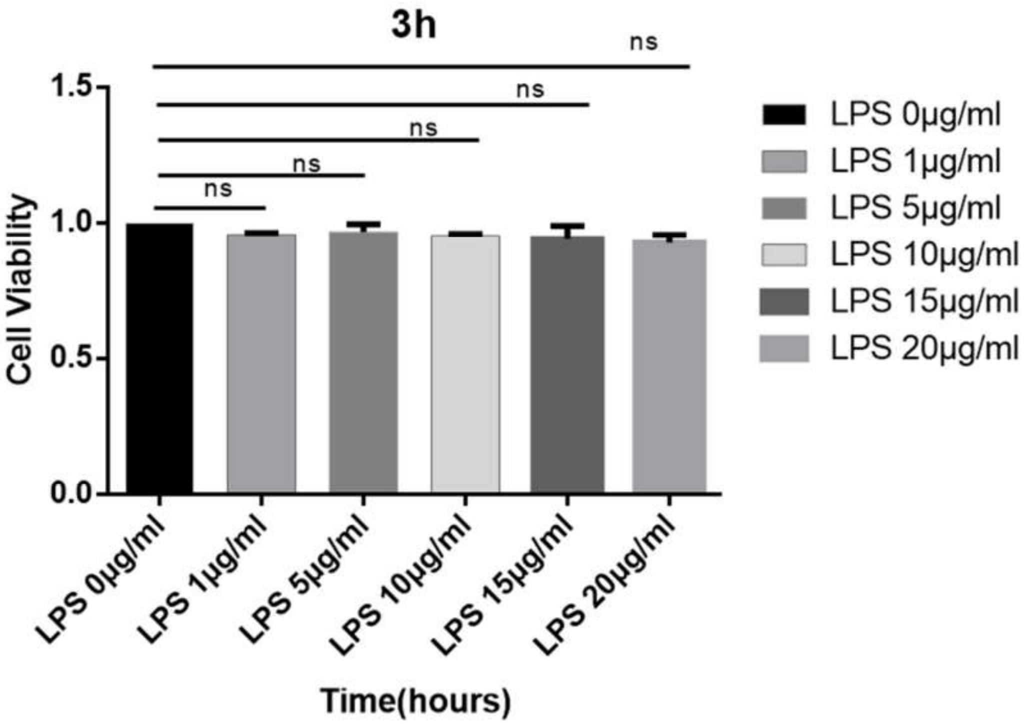

Figure S1. Comparison of LPS effects in 3h group.

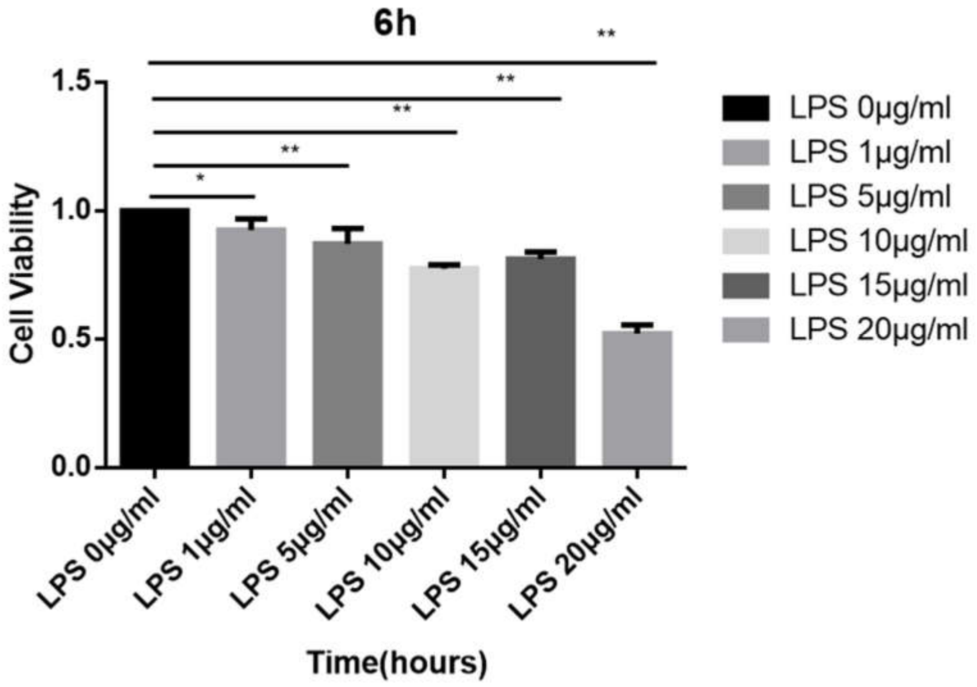

Figure S2. Comparison of LPS effects in 6 h group.

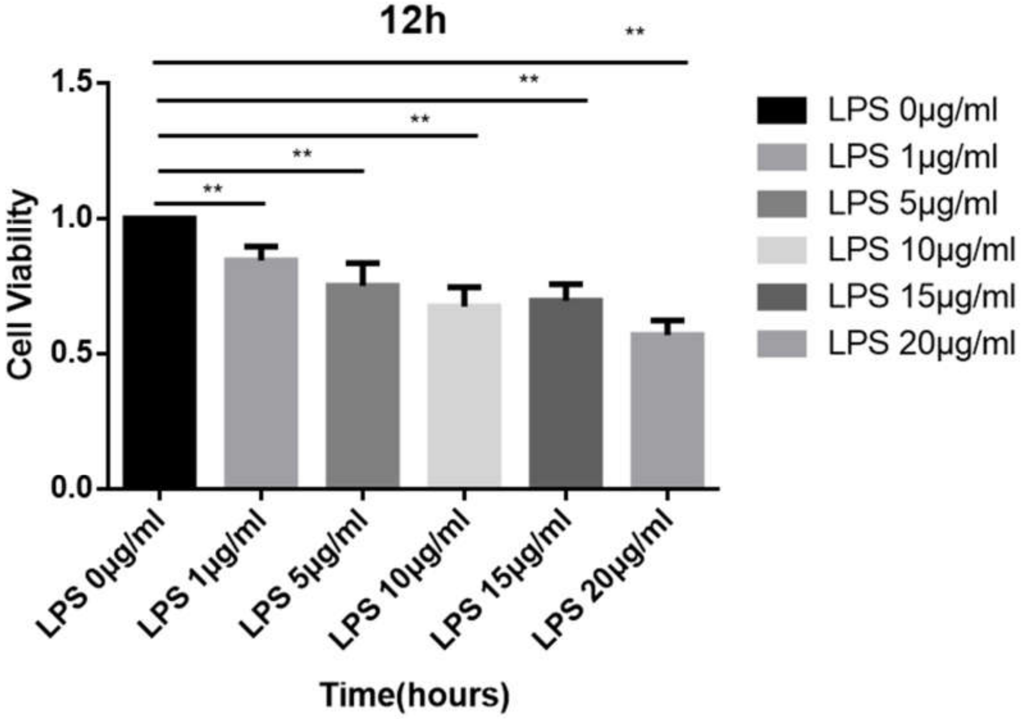

Figure S3. Comparison of LPS effects in 12 h group.

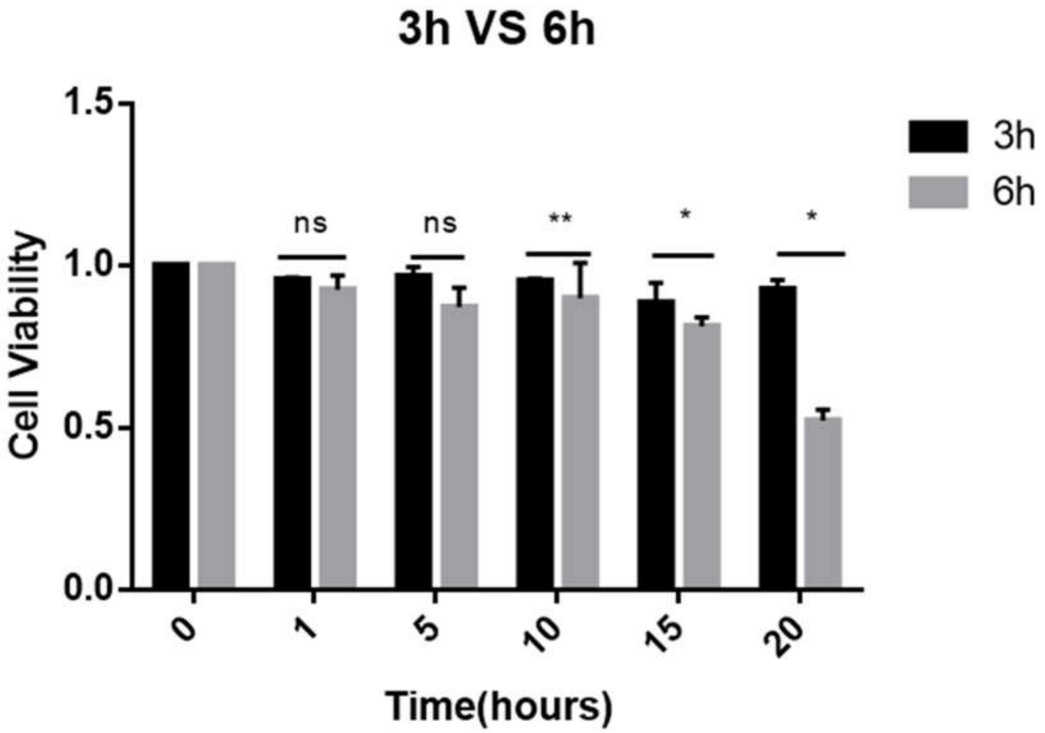

Figure S4 . Comparison of LPS effects between 6h and 12h groups.

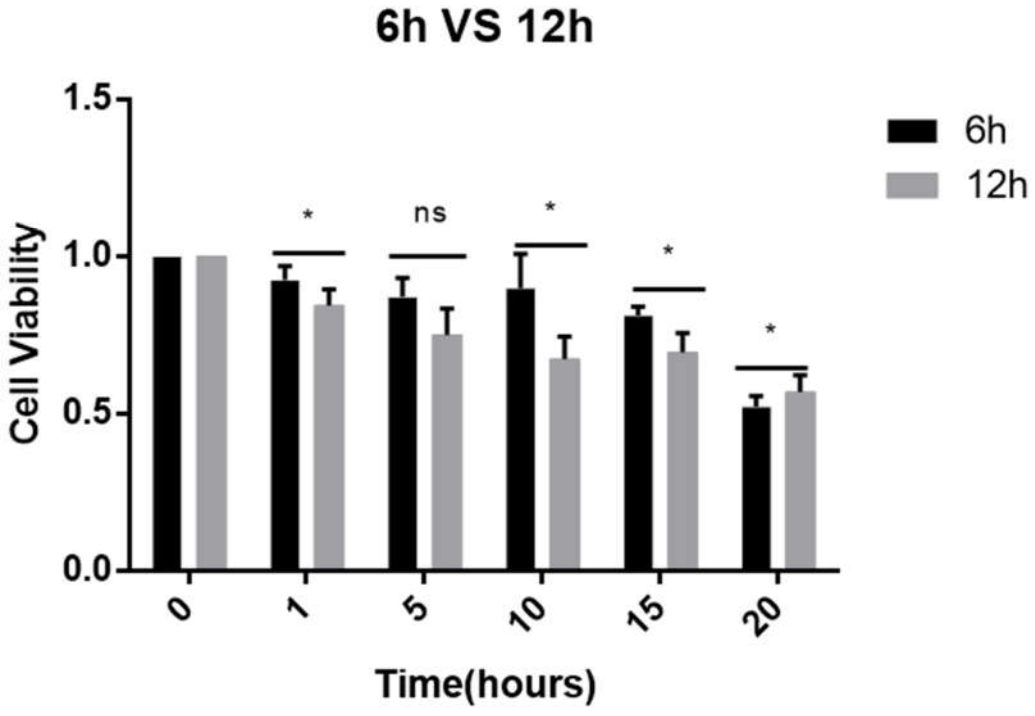

Figure S5 . Comparison of LPS effects between 6h and 12h groups.

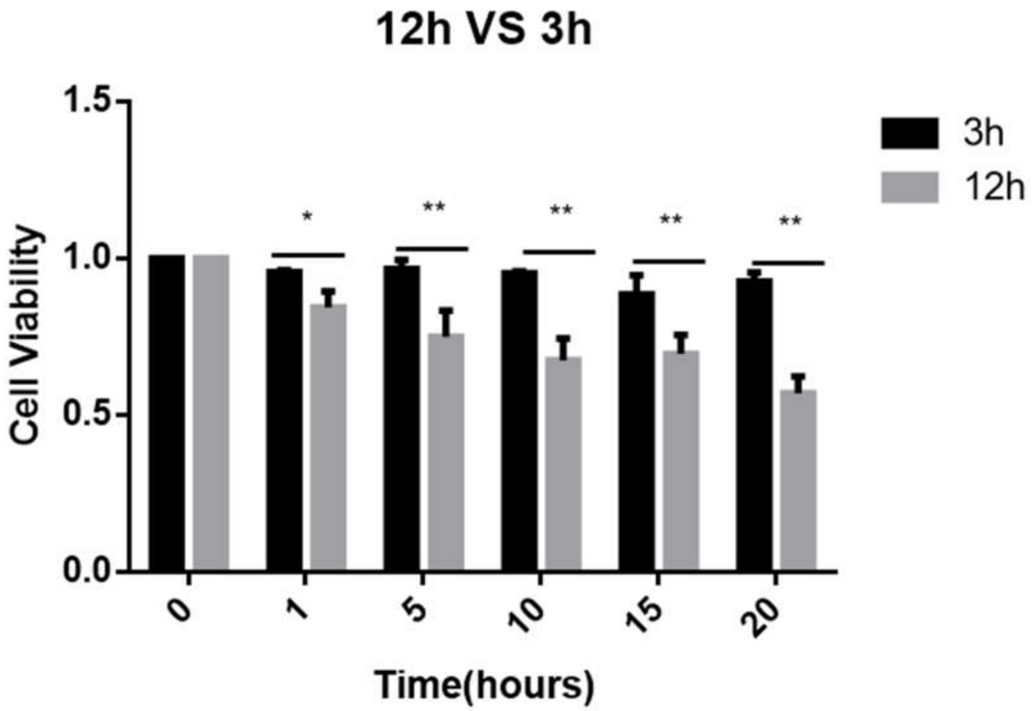

Figure S6 . Comparison of LPS effects between 12h and 3h groups.
